# Supplementary material for: Spin-dependent scattering induced negative magnetoresistance in topological insulator Bi2Te3 nanowires
Source: Sci Rep. 2019 May 24;9:7836. doi: 10.1038/s41598-019-44265-5 (PMC6534536; doi:10.1038/s41598-019-44265-5)
Supplement: Supplementary file 1 — Supplementary info [file 41598_2019_44265_MOESM1_ESM.pdf]

Supplementary Materials

Spin-dependent scattering induced negative magnetoresistance in topological insulator Bi_2Te_3 nanowires

Biplab Bhattacharyya,[1, 2] Bahadur Singh,[3] R. P. Aloysius,[1, 2] Reena Yadav,[1, 2] Chenliang Su,[3] Hsin Lin,[4] S. Auluck,[2] Anurag Gupta,[1, 2] T. D. Senguttuvan,[1, 2] and Sudhir Husale,[1, 2] *

[1] Academy of Scientific and Innovative Research (AcSIR), National Physical Laboratory, Council of Scientific and Industrial Research, Dr. K. S Krishnan Road, New Delhi-110012, India.

[2] National Physical Laboratory, Council of Scientific and Industrial Research, Dr. K. S Krishnan Road, New Delhi-110012, India.

[3] SZU-NUS Collaborative Center and International Collaborative Laboratory of 2D Materials for Optoelectronic Science & Technology, Engineering Technology Research Center for 2D Materials Information Functional Devices and Systems of Guangdong Province, College of Optoelectronic Engineering, Shenzhen University, ShenZhen, 518060, China.

[4] Institute of Physics, Academia Sinica, Taipei, 11529, Taiwan.

*E-mail: husalesc@nplindia.org

Contents:

1. **Elemental characterization of the Bi_2Te_3 nanowire device**

1. Elemental characterization of the Bi_2Te_3 nanowire device

In order to have better understanding of the content of Ga in the Bi_2Te_3 nanowire and in the surroundings of the nanowire, here we provide the elemental characterization of our sample using energy-dispersive X-ray spectroscopy (EDS).

Figure S1 depicts the elemental composition in the Bi_2Te_3 nanowire (spectrum 1) and in the FIB milled region (spectrum 2). As expected in the Bi_2Te_3 nanowire region (spectrum 1), we can clearly see the EDS peaks for Bi, Te and Ga (Fig. S1c). The atomic % of Ga in this region is 4.61%. As expected in the milled region (spectrum 2), we can see the EDS peaks for Si, O and Ga (Fig. S1d). Si and O atoms are due to the Si/SiO2 substrate used in this study. Atomic % of Ga in the FIB milled region is 4.4%. In order to check whether there is any Bi material in the milled region, we deliberately added a Bi peak in the EDS spectrum (Fig. S1d) for which the atomic % was estimated to be 0% (as expected).

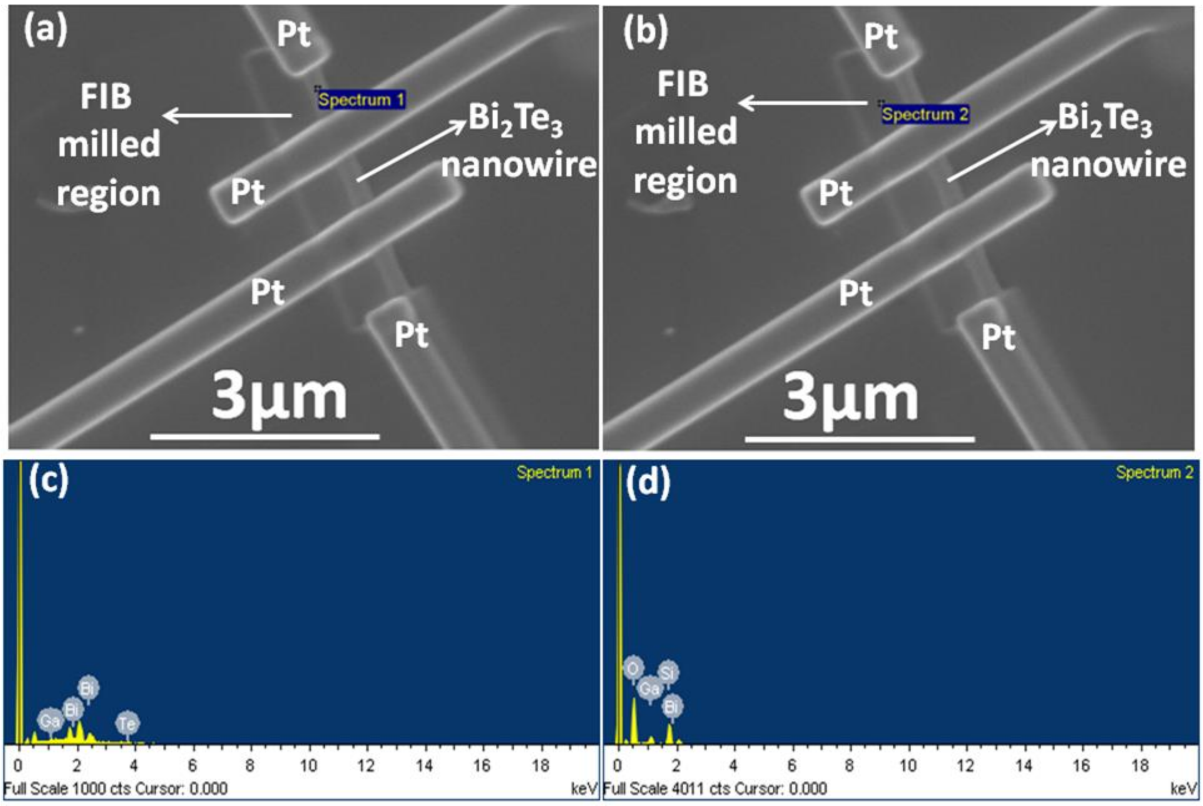

Fig. S1. (a, c) FESEM image and EDS spectrum (#1) of Bi_2Te_3 nanowire. (b, d) FESEM image and EDS spectrum (#2) of the milled region. The stickers named Spectrum 1 (a) and 2 (b) depict the area where elemental analysis was performed.

Figure S2 depicts higher magnification FESEM images of the device, where the elemental composition in the channel length of Bi_2Te_3 nanowire (spectrum 10) and in the un-milled region (spectrum 11) is shown. As expected in the Bi_2Te_3 nanowire region (spectrum 10), we

can clearly see the EDS peaks for Bi, Te and Ga (Fig. S2c). The atomic % of Ga in this region is 2.33%. For the un-milled region (spectrum 11), we can see the EDS peaks for Si, O and Ga (Fig. S2d). Si and O atoms are due to the Si/SiO2 substrate used in this study. Atomic % of Ga in the un-milled region is 0.06%, which is very less as no Ga ion milling has been performed in this region. Again, in order to check whether there is any Bi material in the milled region, we deliberately added a Bi peak in the EDS spectrum (Fig. S2d) for which the atomic % was estimated to be 0% (as expected).

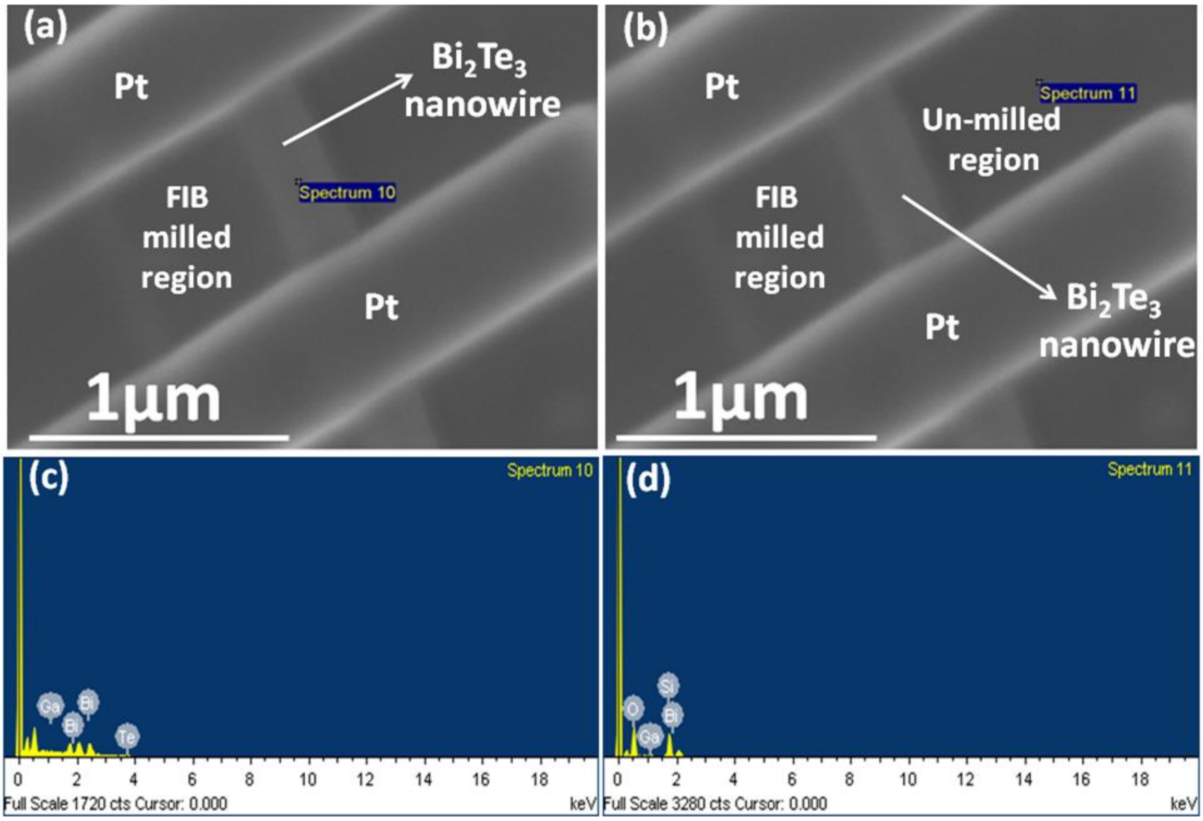

Fig. S2. (a, c) Higher magnification FESEM image and EDS spectrum (#10) of Bi_2Te_3 nanowire. (b, d) Higher magnification FESEM image and EDS spectrum (#11) of the un-milled region. The stickers named Spectrum 10 (a) and 11 (b) depict the area where elemental analysis was performed.
